# “*Kala-Azar is a Dishonest Disease*”: Community Perspectives on Access Barriers to Visceral Leishmaniasis (Kala-Azar) Diagnosis and Care in Southern Gadarif, Sudan

**DOI:** 10.4269/ajtmh.17-0872

**Published:** 2018-02-26

**Authors:** Temmy Sunyoto, Gamal K. Adam, Atia M. Atia, Yassin Hamid, Rabie Ali Babiker, Nugdalla Abdelrahman, Catiane Vander Kelen, Koert Ritmeijer, Gabriel Alcoba, Margriet den Boer, Albert Picado, Marleen Boelaert

**Affiliations:** 1 Institute of Tropical Medicine, Antwerp, Belgium;; 2 Médecins Sans Frontières Campaign for Access to Medicines, Geneva, Switzerland;; 3 Faculty of Medicine, Kala-Azar Research Center, University of Gadarif, Al Qadarif, Sudan;; 4 Médecins Sans Frontières, Amsterdam, The Netherlands;; 5 Médecins Sans Frontières, Geneva, Switzerland;; 6 KalaCORE Consortium, London, United Kingdom;; 7 ISGlobal, Barcelona Institute of Global Health, Barcelona, Spain

## Abstract

Early diagnosis and treatment is the principal strategy to control visceral leishmaniasis (VL), or kala-azar in East Africa. As VL strikes remote rural, sparsely populated areas, kala-azar care might not be accessed optimally or timely. We conducted a qualitative study to explore access barriers in a longstanding kala-azar endemic area in southern Gadarif, Sudan. Former kala-azar patients or caretakers, community leaders, and health-care providers were purposively sampled and thematic data analysis was used. Our study participants revealed the multitude of difficulties faced when seeking care. The disease is well known in the area, yet misconceptions about causes and transmission persist. The care-seeking itineraries were not always straightforward: “shopping around” for treatments are common, partly linked to difficulties in diagnosing kala-azar. Kala-azar is perceived to be “hiding,” requiring multiple tests and other diseases must be treated first. Negative perceptions on quality of care in the public hospitals prevail, with the unavailability of drugs or staff as the main concern. Delay to seek care remains predominantly linked to economic constraint: albeit treatment is for free, patients have to pay out of pocket for everything else, pushing families further into poverty. Despite increased efforts to tackle the disease over the years, access to quality kala-azar care in this rural Sudanese context remains problematic. The barriers explored in this study are a compelling reminder of the need to boost efforts to address these barriers.

## INTRODUCTION

In eastern Africa, inadequate access to early diagnosis and treatment is a critical barrier to the control of visceral leishmaniasis (VL). Despite a decline in global estimates, the region continues to report high and gradually increasing number of cases.^1,2^ Visceral leishmaniasis, also known as kala-azar, is almost always fatal without timely treatment.^3^ Delays in diagnosis and treatment not only increase the risk of morbidity and mortality, but also the risk of transmission of infection to others.^4^ Visceral leishmaniasis control has been hampered by poorly functioning health services, but, on the other hand, increased supply does not always guarantee optimal uptake of services nor impact on the epidemiological trend.^5,6^ Health-seeking behavior toward kala-azar in these sparsely populated rural and underserved areas is complex.

Visceral leishmaniasis is caused by intracellular protozoa from the *Leishmania* species and transmitted by bites of a Phlebotomine sandfly. Its symptoms—prolonged fever, loss of appetite, and spleen enlargement—may mimic other diseases such as malaria, typhoid fever, tuberculosis, or brucellosis. Malnutrition, poverty, and immunodeficiency are known risk factors for developing kala-azar disease,^7,8^ and civil unrest, migration, and severe food shortages have led to large VL epidemics in the past.^9,10^ The disease disproportionately affects the poor and marginalized, and, in a vicious circle, pushes the affected families into further destitution.^11,12^

Sudan bears one of the highest VL burdens in the world reporting 2,000–7,000 cases per year.^13^ Visceral leishmaniasis is thought to be primarily anthroponotic here.^14^ Vector control strategies for VL include insecticide spraying, use of insecticide-treated materials, and environmental management.^15^ Unfortunately not much evidence exist about their effectiveness in eastern Africa and they are not widely used. In the absence of vaccines and effective vector control strategies, case detection and treatment remains the principal VL control approach. The national VL control program is in place for more than 10 years, providing diagnosis and treatment at public hospitals as a main strategy.^16,17^ Diagnosis relies on an antibody detection test, the direct agglutination test, or rK39-based rapid diagnostic test (RDT), or on parasitological examination.^18^ Since 2011 the first-line treatment is 17-day injections of antimonial and paromomycin,^19,20^ requiring hospitalization for part or the entire course. Late presentations to the hospitals are common, especially in a predominantly rural area such as Gadarif.^21^

Gadarif state of eastern Sudan contributes to 80% of the number of VL cases reported in the country.^22^ The southern part of Gadarif is a highly endemic zone with an incidence rate of 75 cases per 10,000 persons per year in a village-level study in 2012.^23^ Not all VL patients present themselves to the hospital, although the underreporting seems to improve in recent years.^24,25^ Over the past 30 years, several nonstate actors provided support to the Ministry of Health (MoH) to tackle VL, yet the number of cases remains high and access to VL care remains a critical issue in this region. In these large expanses of hard-to-reach, isolated areas there are limited number of hospitals where VL can be treated. During the rainy season (May–October), many roads are impassable, and subsistence farmers and laborers will prioritize the agricultural calendar and postpone dealing with any health matters during these months.^26^ Although the governmental hospitals offer VL treatment free of charge to patients, other costs such as transport, registration, admission, drugs for concomitant diseases, and laboratory tests are not for free.^27^ Drug shortages have been observed in the MoH services, further reducing access to treatment of many. Cultural barriers exist as well. Nomadic groups lack awareness of the disease. Restrictions on women’s decision-making power and use of traditional remedies have also been identified as access barriers in a study conducted 13 years ago.^28^ All these barriers taken together may result in a long delay between the onset of symptoms and treatment, complicating VL case management, and the chance of treatment success. Treatment defaulters and losses to follow up are common, and the reasons behind are poorly understood.^26^ Most interventions in the last decade, however, have been focusing on the supply side of the health service by opening more VL treatment centers, and supplying them with RDTs and medicines. The reasons behind the continued stagnation/increase in the number of reported VL cases, even across villages with similar conditions, are still largely unknown.^29,30^ The perspectives of the people themselves, as end users, are rarely investigated.

This qualitative study aimed to explore the perceptions and attitudes of the community to understand the barriers in accessing kala-azar care in this setting. A better understanding of the social context of kala-azar in an endemic area such as Gadarif would generate insights on practical ways to enhance access to care and adjust future control activities.

## METHODS

### Conceptual framework.

In this study, we consider “access” in terms of whether those who need kala-azar care get into the care system and what factors impede this access. We initially adopted the three-delays model from Thaddeus and Maine (delay in the decision to seek care, delay in getting to the facility, and delay in obtaining appropriate care once at the facility)^31^ and further incorporate the health behavior model of Andersen^32,33^ that focuses on utilization of health services. This model aims to explain use of health services as a function of a set of predisposing factors, enabling/disabling factors, and need factors. This framework guided the structuring of our findings into individual-, population-, and health-system levels barriers that influence health-seeking behavior toward kala-azar.

### Study setting and population.

Gadarif state has a total population of 1.4 million, spread over 75,000 km^2^.^34^ It is ethnically very diverse—many Arabic, western Sudanese, West African, and non-Sudanese tribes settled there during the agricultural boom in the 1960s.^35,36^ The vegetation consists of a typical dry savannah woodland, with *Acacia* and *Balanites* trees, combined with black cotton soil. Agriculture is the main livelihood, with sorghum, sesame, and millet as major crops. More than half of the population live in rural areas, and only 60% are literate.^37^ Most people are subsistence farmers or engage in small animal husbandry. Socioeconomic inequalities are high, due to the expansion of large mechanized farming based on underpaid wage labor. High demand for manual labor attracts seasonal workers (from within Sudan or bordering Ethiopia) during the rainy season.

**Figure 1.**
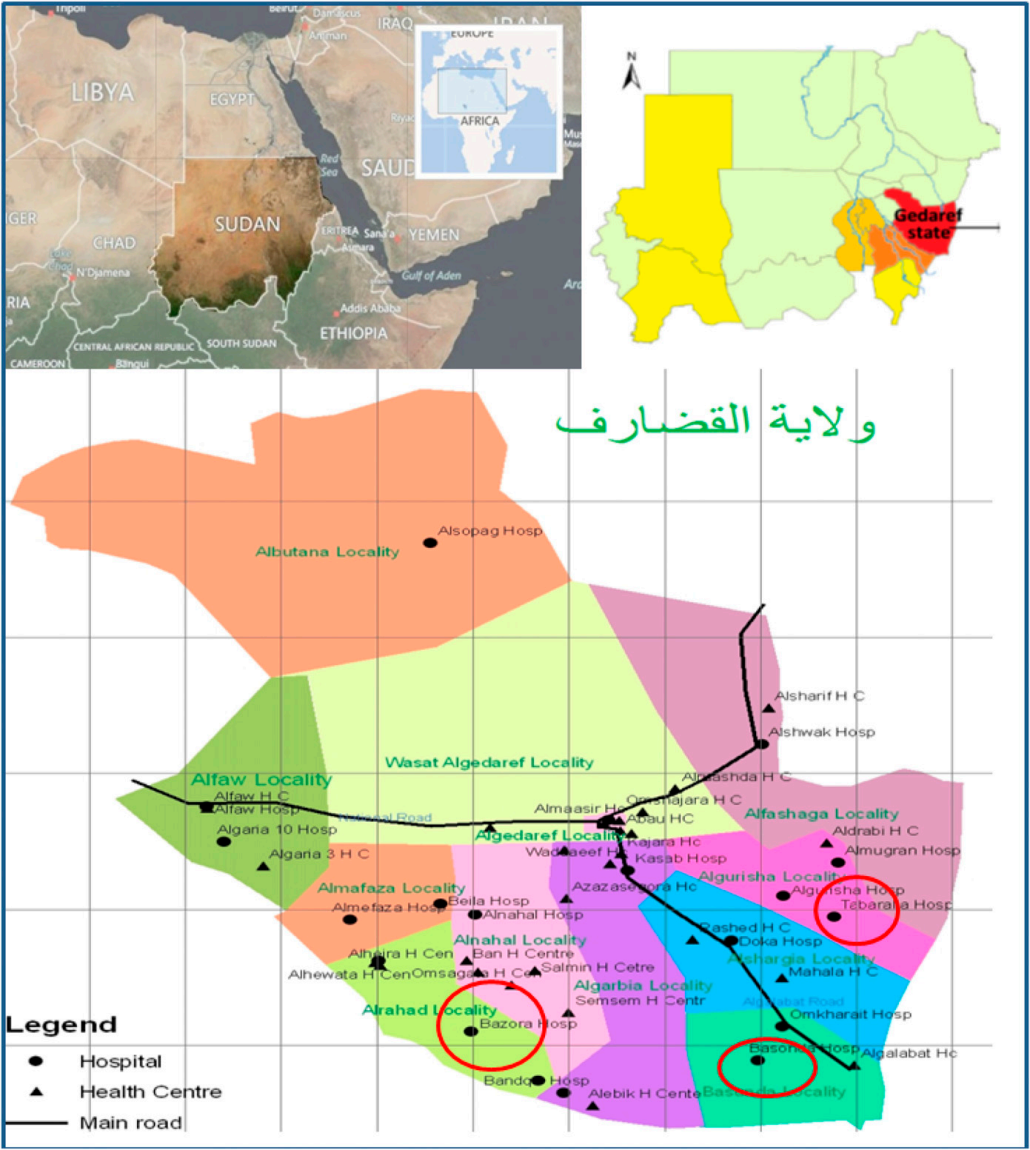
Map of Gadarif state in eastern Sudan and localities where the study is conducted. This figure appears in color at www.ajtmh.org.

The study was conducted in three most VL–endemic localities in southern Gadarif – two located along the Atbarah and Rahad river basins (Qureisha and al Rahad, respectively) and one directly bordering Ethiopia (East Galabat) (Figure 1). Al Rahad locality is served by two hospitals, Um el Kher (supported by Médecins Sans Frontières [MSF] in 1996–2005) and Bazoora (also supported by MSF starting in 2017). East Galabat locality, is served by one rural hospital (Basunda) and one specialized kala-azar center (Doka) supported by the research agency Drugs for Neglected Disease Initiative). The al Qureisha locality is served by a kala-azar center supported by MSF since 2009 (Tabarak Allah).

Historically, MoH was in charge of and providing kala-azar care in Gadarif. When a VL outbreak unfolded in 1995–1996, efforts to study and tackle the disease increased, which included collaborations between the Sudanese and international actors. non-governmental organizations (NGO) as MSF have treated thousands of VL cases in dedicated clinics. More recently, the KalaCORE consortium is supporting MoH to improve routine kala-azar services since 2015. In 2017 there were 10 public hospitals designated to diagnose and treat kala-azar, including the referral hospital in Gadarif town.

### Study design.

We conducted a qualitative study combining in-depth interviews (IDI) with individual key informants and focus group discussions (FGD) to add breadth to the data and triangulate the findings.^38^ Data collection took place in March 2017. The field research team was composed of equal numbers of members with biomedical and social science backgrounds, four females and four males, and supervised by the principal investigator (T. S.). We purposively selected three villages from each locality, one with a high number of cases in the last year, one with low number, and one with the worst physical access to any kala-azar treatment centers in terms of roads. We decided on this choice of villages in two meetings between the research team and community facilitators from the localities, using village level kala-azar data from state MoH. The purposive sampling was chosen to ensure maximum depth and variation of information, including typical and deviant cases. Participants for IDIs consisted of three categories: former kala-azar patients or caretakers (as community member), community leaders, and health-care provider. Participants for FGDs consisted of community members only, as involving community leaders or health workers in the discussions may introduce bias.

We introduced the study and the aims to local village chiefs, and community facilitators who previously invited participants for FGDs further approached key informants for a face-to-face interview. If participants were willing to participate, the research team visited them in their home and obtained a written consent before conducting the IDIs. Group verbal consent was taken before FGDs. Health workers in the area were consulted but to avoid bias as much as possible the IDIs and FGDs were not conducted in the health centers. Between 7 and 12 people participated in each FGD which lasted 45–60 minutes, whereas the IDI lasted on average between 40 and 90 minutes.

Focus group discussions were conducted separately for women and men. The interviewer/moderator was of the same gender as a participant(s). All interviews and FGDs took place in the participants’ homes or other private and confidential areas and were, after permission, recorded on a digital voice recorder. Semistructured topic guides were used to guide the IDIs/FGDs, and additional items were included as data collection progressed. Data collection continued until saturation was reached and no new information emerged. All interviews were conducted in *Arabic*, the main local language.

### Data management.

To ensure quality of data, we put forward the following mechanisms during data collection: 1) permanent supervision of the study by the principal investigator (T. S.) and 2) frequent exchange and feedback sessions among the study team in the field. Before study implementation, the team completed a 3-day training workshop, after a pilot test of study tools in one village nearby Gadarif town. Records from the study were transcribed verbatim, while an Arabic language expert and native speaker (G. K. A.) supervised the data transcribing and translation process. The subsequent English translation was carried out by a team from a local English language institution and cross-checked by two researchers (R. A. B. and N. A.).

A public health specialist (T. S.) and anthropologist (C. V. K.) independently analyzed the data using a thematic analysis approach.^39,40^ Themes were identified through careful reading and re-reading of the data, and if patterns were recognized, these emerging themes became the categories for analysis. This analysis method combines a deductive approach - through predefined themes in the questions guide- and data-driven inductive approach allowing for themes to emerge from the data. Three researchers (T. S., C. V. K., G. K. A.) conducted the refinement and reconciliation of coding through frequent discussions about deviations and common themes. All team members were involved in revising the coding schemes, organizing the codes and corresponding quotes to identify consistencies and contradictions in the data and interpretation. NVivo software (version 11; QSR International, Melbourne, Australia) was used to aid the data management during analysis.

### Ethical statement.

We obtained ethical approval from the National Health Research Ethics Review Committee, Federal MoH of Sudan and from Institute of Tropical Medicine Ethical Review Board. Permissions to conduct the study were obtained from the State MoH, local authorities, and MSF. Written informed consent was obtained from each study participant or their parent/guardian for the nonadult participants, in addition to their assent. Additional oral consent was obtained for using digital voice recorder. Participation in the study was voluntary, and any information provided was kept confidential. Quoted information was anonymized during the analysis and reporting.

## RESULTS

A total of 191 individuals participated (see Table 1).

**Table 1:** Characteristics of the participants of IDI and FGD

		Females	Males	Total
**In-depth interviews**		**10**	**14**	**24**
Age group	14–25	2	0	2
	26–35	2	5	7
	36–45	4	2	6
	46–65	3	6	9
Categories	Community members*	8	4	12
	Community leaders†	1	4	5
	Health-care workers	5	2	7
**Focus group discussions**		**9**	**10**	**19**
Locality	Rahad	4	5	9
	Qureisha	2	2	4
	East Galabat	3	3	6

FGD = focus group discussions; IDI = in-depth interviews.

* Community members: former kala-azar patients or caretaker of a patient.

† Community leaders: members of people’s committee, school principals/teachers, merchants, or religious leaders.

### Kala-azar is well known, yet with varying level of misconceptions.

Kala-azar is a well-known illness and familiar to many, as almost all participants knew someone who suffered from it. The history of VL goes back a long time in Gadarif. Respondents told that the disease was previously known under the local name “*maraad sayeed*,” literally meaning “disease of the south”—referring to the upstream river basin areas. Other vernacular synonyms for kala-azar were “*tohaal*” (abdominal swelling), “*suffair*” (jaundice), and “*ghibia*” or “*jini wardah*” (recurring fever). Our respondents describe the disease as characterized by bouts of fever, abdominal swelling, low appetite, and weakness. People also linked kala-azar with vomiting, headache, yellow skin, pale eyes, nose bleeding, dry lips, and anxiety.

Respondents claimed that the name “kala-azar” was only introduced when “outsiders” came to Gadarif to investigate the “killing disease” outbreak in the 1990s. These outsiders were doctors and researchers from Khartoum and abroad and they were the ones who told the villagers about a “sandfly” causing this disease. Respondents from the villages along the Rahad and Atbara rivers were mostly able to correctly identify the sandfly as the transmitting agent, and its association with the *lalob* (*Balanites aegyptica*) and *taleh* (*Acacia seyal*) trees, cracks of the soil and the mud huts, and animal dirt. However, some participants did not know what causes kala-azar and attributed it to hunger, contaminated water, mosquitoes, unhygienic houses, and staying outside the village on the farmland. Some participants believed that the disease could be passed from person-to-person in the same house. Drinking water or sharing food with patients, sleeping in the same bed, and clothes and sweat of the patients were also evoked by some as ways to get infected.

*“Because when you eat or drink some of the food or drinks meant for the sick person, you will be infected. When this disease infects a person in a family, it must also infect two or three other persons”* [IDI, Male, Community leader]

Some people said that everyone, rich and poor alike, can get kala-azar. However they acknowledged the vulnerability of some groups: children, or the young, in general, were thought to be more at risk, as they play outside close to the trees where the sandfly lives and have “weaker blood.” Family members and poor families with food insecurities are also perceived to be at a higher risk to get kala-azar. Certain areas were thought to be more at risk: villages along the river and the deep remote settlements far from the road. A few participants hinted that certain tribes have “more kala-azar”: the Hausa and Masaleet were mentioned; although for most respondents the geographic location of their villages matters more. Most respondents linked the abundance of sandflies with the increased number of kala-azar after the rainy season/autumn, also known as the “sesame season.” Several mentioned that the general fatigue that people experience after harvesting, makes it easy to contract kala-azar.

*“Yes, hunger causes it, the sandfly brings it. The person who does not eat enough definitely he will be sick and the illness will increase. When the fly finds that your blood is weak, it (kala-azar) strikes you”* [FGD, Female, Kersyba]

### Diagnosis delay and multiple trajectories (getting diagnosed is not an easy feat).

Participants who had kala-azar in the past or cared for family members with kala-azar claimed that the search for a definitive diagnosis is a major challenge. People first try to self-medicate, adopting a “symptomatic” approach with distinct healing methods for each affliction. For example, they rub oil and herbal paste against fever, cut or burn skin against the abdominal swelling, drink herbal concoctions from various roots for yellow skin, or drink water that has been blessed by readings from Qur’an (*muhaya*) as a more general measure. The association with “bad blood” led to the practice of bloodletting, “letting the black blood run,” after which the wound is tied up with tree barks. If these attempts are unsuccessful, then only people resort to the formal health system—this might be the village health center or clinic, nearest rural hospital, or pharmacies, depending on circumstances and available money. Private clinics are rare except in Gadarif town.

However, many participants said that the traditional treatment is a thing of the past, from the time when the kala-azar drug was neither known nor available. These traditional or religious practices still have their place if the kala-azar test results are negative. Traditional healing practice seems ubiquitous, not only in remote villages. It is also enhanced by the financial barriers linked to the formal health system.

*“When we do not know, we treat him traditionally. If not healed, we take him to the doctor to inject him, (but) to take the injection he needs to be tested first. When you go the doctor, regardless if kala-azar appears or not, the doctor gives an injection for malaria and inflammation. If this fails, then we need to take him to the doctor for (another) testing.”* [IDI, Female, Former kala-azar patient]

*“… I have seen it for a long time that local medicine does not cure kala-azar. What is cured by local medicine is “Um-Siffair” - jaundice – trees can cure that. However, kala-azar needs kala-azar medicine. Sometimes, it needs some local medicine to come out and be found. This is the case when it does not show at first in the laboratory test.”* [IDI, Male, Former patient]

People mostly believed that kala-azar can only be found after multiple tests. The “easy” type of kala-azar is the one that is detected immediately, whereas the “difficult” one is the one with repeated negative test results. Kala-azar is to a large extent thought to be “hiding” in the body and will only show itself after evolving from malaria, inflammation, or typhoid. In the experience of many, there is a need to be treated for other diseases first.

*“We say it is malaria first and then it changes into kala-azar. Sometimes they say typhoid. Malaria becomes typhoid and typhoid becomes kala-azar”* [FGD, Male, Rymila]

*“Kala-azar is a dishonest disease. If there is any other disease in your body, then kala azar will not appear in the test until you get rid of all the diseases you have.”* [FGD, Male, Tabarak Allah]

Most participants indicated that if they suspect kala-azar, they will seek to confirm this through repeated investigations; especially in a bigger town such as Gadarif, where a private laboratory, in particular, is known to be a trusted kala-azar laboratory in the state. The village-level health center and the clinics only perform RDTs for malaria. Hence many respondents described their experience having to undergo multiple tests in various places, either referred or by their own volition, in search of the final kala-azar diagnosis. When the results turned out negative, several respondents attribute it to the lack of experience of the technician or unavailability of “sophisticated machines.” Health workers said that the RDTs are not reliable and that only by microscopy the diagnosis can be ascertained.

*“We are poor. I have two children who had kala-azar. I went to Gadarif five times. All tests and ultrasound exams did not discover the disease, so they told me to go to Ahmad Daneel (the private kala-azar lab). When he saw them, he said it was kala-azar and transferred me to Doka hospital. When I came, they told me to pay 12 pounds for tablets, and I paid, but they gave me nothing. Waited for another day and again nothing. Then I went to Tabarak Allah center, and they started the tests again, my first child and then his brother was found positive but they recovered, and they are well”* [FGD, Male, Rymila]

Participants also discussed specific food items that are believed to force kala-azar to appear, such as banana, chicken, and fish. Some respondents said that this widespread belief was initially promoted by the doctors, although health-care workers refuted this. According to our respondents, this food would make the symptoms worse and push the kala-azar to appear.

*“Sometimes, when it is too difficult to find kala-azar in laboratory tests, the sick person is told to go home and eat bananas.” …”* [IDI, Male, Former patient]

When kala-azar is finally diagnosed, this is seen as a blessing or good luck. People congratulate the patient with the positive test. People widely felt relieved when kala-azar is diagnosed, as they assume that now cure is within reach and further money expense can be avoided.

*“The demon accompanying kala-azar is the fact that the disease does not appear easily. So when you meet the doctor for diagnosis, you may feel tired and exhausted. You have nothing at home. Before you do the test, they say ‘your medicines are this and that’, and ‘we test, you give the money’ You have no money to pay them. Here you feel worried and anxious... Afterwards when kala-azar appears, you say thanks and praise to Allah. This is a blessing.”* [FGD, Male, Bazoora]

The journey to obtain the diagnosis is a huge challenge and these hurdles are evoked as the most important reason to delay coming to the hospital. Treating the initial symptoms with medicines brought from the market is also common and seen as part of the “diagnostic process.”

*“In case someone is sick, he comes here to the hospital and if no kala azar is found [immediately], he goes away and buys the medicine from outside. In reality, he may have kala-azar, yet he buys the medicine from outside and many patients are lost this way. A person's health worsens, and they bring him here when it is too late.”* [IDI, Female, Health care worker]

### Variable quality of care in different treatment centers.

The irregular availability of the kala-azar medicine at the hospital was a source of concern for many respondents. People who have to wait for the drug linger several days at the hospital, and in some cases are referred to places with a higher likelihood to have the drugs. Several respondents experienced this firsthand, and few mentioned that the medicine could be bought outside the hospital. Universally known as “kala-azar injection,” it seems that people can purchase the vials in an informal circuit if they know someone from “inside” this black market.

*“By Allah, the reason of death is nothing else than unavailability of medicine. Sometimes it is tough for people to find medicine when they are sick with kala-azar, so they die because of lack of treatment. The medicine is not available all the time, and sometimes you will find a person took two or three injections then the medicine is finished.”* [IDI, Male, Healthcare worker, Tabarak Allah]

*“If you know the right local person, you tell him that you need kala azar drug for your son, they tell you ok, we will give you the drug, and the cost is 500 pounds [US$75]”* [IDI, Male, Caregiver]

Most participants said that the injections are the only cure for kala-azar and had no doubt about its effectiveness. However, they also mentioned that the drug is “heavy” and “burns the blood.” The change in the duration of treatment, from 30 days to the current 17 days was noticed and to some, generated a concern that kala-azar relapse happens more often with the new regimen. People also said that there is some food to be avoided to prevent kala-azar relapse or skin spots (referring to the post-kala-azar dermal leishmaniasis), such as Sudanese groundnuts (*foul* or *dakwa*) or beans. Participants from kala-azar–affected households expressed their negative feelings toward the health-care system. They mentioned the poor facilities (such as inadequate bed capacity), lack of medical staff, the commercial motives (everything has its fee), and the variable quality across hospitals. The hospitals that are supported by NGO stand out because all care is provided for free and kala-azar patients receive additional support, thus serve as an incentive for the patients.

*“During the period of treatment they used to give patients soap, flour, and oil for cooking, the sick person gave multivitamin, milk, madeeda (porridge) so when he gets out of the hospital, he was fit.”* [IDI, Male, Former patient]

*“We did two years without a doctor in the normal (not Kala-azar) ward, while MSF managed the Kala-azar center next to it. The organization is very good, but there is a great difference: the governmental hospital is very poor in everything and the government's doctor is paid a weak salary.”* [FGD, Male, Tabarak Allah]

### The perpetual poverty and the taxing journey to reach care.

To deal with a kala-azar episode is costly; this is acknowledged by all IDIs and FGDs participants without fail. Although kala-azar drug is officially provided free of charge, there are many other expenses for the kala-azar patients and their families, which they have to pay out of pocket. These include hospital entry ticket, different laboratory investigations, bed, syringes, medicines for other nonkala-azar conditions, and meals for the caretakers. Participants described their coping mechanisms such as borrowing money, selling cattle/crops, or asking help from the Islamic charity *zakat*. The financial losses are felt both in the short- and long-term, and often stood as the main reason for not taking the sick to the hospital.

*“The financial situation is so bad; you find the families (who) are too poor to have food for tomorrow or even the day, they have no money to see a doctor and buy medicine, so he stays with his disease (kala-azar). They say thank god if recovered, if not then it is God’s will”* [FGD, Male, Um el Kher]

*“It is difficult here to be sick with no money because you need two pounds (US$0.3) to get to the hospital and 20 pounds (US$3) to see the doctor, who will give you three tests, malaria, inflammation or typhoid. The result may not show kala-azar, but he has it. Then you start testing again, and that may cost 60–70 pound$ (US$9–10.5), always need to get back (to the hospital) with the sick - such case is normal here, and when your pounds are finished, there is nothing to give him but the traditional treatment”* [IDI, Male, Community leader]

The transportation cost to get to the hospital varies with season. During the rainy season, transport costs increase significantly as the roads are flooded. The participants described how the sick were carried by stretcher, on boats or on a tractor that may take days or weeks to reach the hospital. Because of this difficulty, many respondents expressed that people would rather wait until the rainy season is over to seek kala-azar care, and will wait even longer, if there are agricultural chores. The unstable income during this period, to some participants, also acts as a deterrent from seeking timely care.

*“Now, in this area for about eight months you [to the interviewer] can't reach us here, I am sure. The tractor was stuck for a day on a stretch where the car can cut in 15 minutes.”* [IDI, Female, Caregiver]

*“First thing is the road, the distance between Barbar and Tabarak Allah is five kilometers, and it takes 6 hours to reach there with the mud…very exhausting. Moreover, people in the rainy season need to buy seed, so they do not have the money to take the patient to Tabarak Allah. From less than 10 pounds (US$1.5) this will now cost 300 to 400 pounds (US$45–60), and you have to rent a special vehicle. Many areas are completely cut-off during the rain. Poor people could not take their son to the hospital even if he was dying in front of them. ”* [IDI, Male, Former patient]

*“The problem is that in autumn, people’s financial situations are very difficult. The patient’s family does not have anything. People collect money for him and write an application to the “Zakat chamber,” and you do not get enough money to help you, it is a complicated situation.”* [FGD, male, Rymila]

### Gender inequalities further exacerbates kala-azar impact on the family.

Kala-azar is broadly perceived as a dangerous disease and should be taken seriously. The notion of danger is linked to its severity and perception that kala-azar ultimately kills or causes death. Most female participants were aware that kala-azar or its treatment can cause abortion.

*“It is the most dangerous disease because it is difficult to be cured. Other diseases can be cured in the nearest hospital but kala-azar only in organizations or centers. It is so expensive, it costs 3000 or 3500 pounds (US$450–525). We think it is the most dangerous disease as it causes death”* [FGD, Male, Barbar el Fugara]

When a family member is suspected to have kala-azar, although both parents are responsible to decide what to do, the woman or the mother is in a disadvantaged position. The women are expected to handle multiple tasks: household chores, agricultural work, and childcare. Permission from the husbands or male family member is culturally required, although this is strongly linked to the financial solvency as the husband’s prerogative. However, respondents also said that women could help raise the money, either by selling crops or reaching to the collective community resources.

*“To get the medicine you have to buy it, so probably sometimes you will not have money, sometimes the father also is not around. A sick child may spend 15 days before going to the hospital, why? The mother tells you that the father was not around and there is no money. She has to wait till the father comes before doing anything. Some people sell their sheep or goat in the market to get money. So money is a major problem, (it) will block everything and that is why there is a delay.”* [IDI, Female, caregiver]

Many female participants spoke of the impact of kala-azar on the family, the emotional toll to deal with anxiety related to the disease and premature death, and further impoverishment to the family, even if the patient is not the main breadwinner. Having to stay in the hospital disrupts their life, as the work in the field has to be abandoned or delegated to other people.

*“The family will be troubled despite their poverty they do what is beyond their abilities. All the family care should be directed to the patient, kala-azar is most dangerous disease here and is still a problem.”* [IDI, Female, Caregiver]

### Limited efforts to control the disease.

Participants from villages with a high number of kala-azar cases in the past indicated that the disease has somewhat “decreased” now, although few mentioned that it might increase again. Most people perceived a change in the situation, attributed to various things such as the presence of “organizations,” health education, and preventive measures, e.g., spraying campaigns, mosquito net distribution, and cutting trees.

*“In the past, kala-azar was more common here, but now and after the organization came the disease is reduced. The people come to our area from different regions to get treatment here. The deaths are also much less. The people nowadays use nets, they know how to control the disease, and there is health education for us. Previously, there were only grass and bushes which are a good environment for the fly.”* [IDI, Female, Health care worker]

The preventive measures, however, are not entirely perceived as successful. Both the villagers and health-care workers expressed their weariness, as to the use of mosquito nets (too hot in summer time), the ineffectiveness of spraying, and the abundance of trees, even if they are permitted to cut them.

*“We are worried about the cause of the disease and treatment. However, we keep asking how to fight the sandfly? From our experience to cut down trees and run after the sand fly to catch it, [everybody laughs] this is a failure.”* [FGD, Male, Tabarak Allah]

When asked about what more could be done to tackle the disease, many participants expressed that more centers are needed for the hard-to-reach areas, including more laboratory and more doctors. Although some participants wished that kala-azar–specific activities such as spraying, net distribution, and health education programs (in the mosque or school) would be improved, others say that economic programs to tackle poverty would be more critical. Many respondents spoke of the need for more roads and better transportation in autumn.

*“I would say that the State should help citizens, treat them & deal with the things that are difficult for them to handle. “The poor citizen cannot get medicine and searches for the cost of treatment.”* [IDI, Male, Community leader]

## DISCUSSION

**Figure 2.**
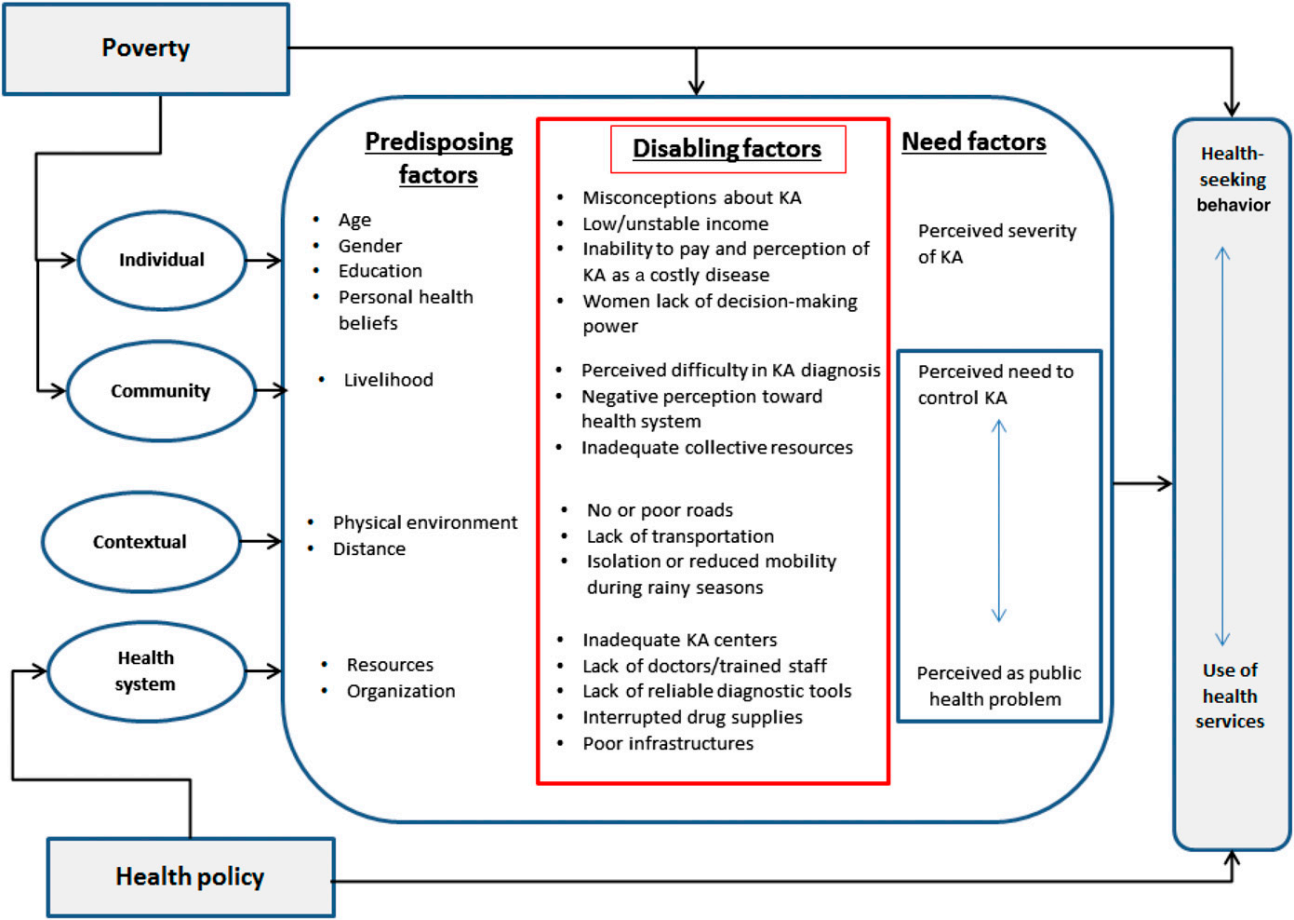
Summary of access barriers to kala-azar care in southern Gadarif, Sudan. This figure appears in color at www.ajtmh.org.

This qualitative study explored the barriers to access kala-azar care in endemic areas in southern Gadarif, Sudan, from the perspective of the people. Our findings describe the multitude of difficulties people face when seeking kala-azar care, and illustrate the prevailing hardship in a rural Sudanese context. The various barriers, as experienced and narrated by study participants, are depicted in Figure 2. Access to health care is always a multidimensional phenomenon closely related to the health-seeking behavior of the population. However, in this region the perception of illness and care is predominantly shaped by poverty and other structural problems in an extremely resource-constrained setting.

Both the three-delays and health behavior models have been used in many health topics,^41–44^ yet this is the first time to apply them to the context of kala-azar in Sudan. Our study was designed to explore what barriers persist from the community perspective on kala-azar care. The predisposing characteristics at community and health system levels influence the “propensity” of individuals to seek care (stage of the decision as the first delay). The second delay in reaching health care is closely linked to the contextual elements, and the third delay in getting quality care is related to the barriers at the health system. Several barriers that we identified were comparable to findings in a community-based study in the same area^28^: lack of money for treatment and transport, distance, impassability of roads, work priorities, and gender inequality. A more recent study in 2012^23^ reported that the population around Tabarak Allah (where MSF runs a kala-azar center since 2009) have adequate access to care, but our findings show that this is not necessarily the case elsewhere. Geographical and financial barriers are essential,^11,45–47^ and the situation is more complicated than assuming that increasing the number of hospitals would be the solution.^48^

In this study, participants were well aware of kala-azar as an important communicable disease, as have been reported in other kala-azar endemic settings.^49–51^ The cardinal symptoms are known, although misconceptions of the disease regarding its real cause and transmission remain. The knowledge on sandfly, its habitats in certain trees or cracks were translated to prevention efforts: vector-specific (bed nets, spraying, cutting trees, repellant oil, etc.) or general ones such as improving hygiene. As kala-azar seems to persist in the community; there is a sense of eroded trust in the preventive measures promoted by health providers. The perceived severity of kala-azar is the main drive behind the continuation of such practices, despite the lack of evidence of effective vector control in this area.^15,52,53^

We did not find local explanatory models of disease^54^ that are incongruent with the biomedical knowledge that has spread along with efforts to control the disease. None of the participants explained kala-azar as related to the supernatural, magical or religious factors, indicating that the prevailing belief is of naturalistic causality.^55^ When asked about the use of traditional medicine, people unanimously said that these things are not for kala-azar. However, people also described these traditional practices for several kala-azar–associated symptoms, such as the swollen spleen (*tohaal*) with burning or jaundice with herbal remedies. Therefore, despite a relatively high level of knowledge about the disease, this may not necessarily mean that there is no “shopping around” for treatment. The study by Gerstl et al.^28^ described similar behavior, although they found that disease awareness was low. The care-seeking itineraries for kala-azar are not straightforward; they appear to depend on the symptoms, perceived cause, duration, severity, and role of the person in the households.

Diagnosing kala-azar is seen as a critical difficulty for the people, as the gateway to treatment is only through a positive diagnostic test. Kala-azar is perceived as a disease that is in hiding. Thus various examinations are required, and other diseases must be treated first—most notably malaria and typhoid. There seems to be a low awareness on the type of the tests that is adequate to diagnose kala-azar. Gadarif is an unstable seasonal malaria area, and concomitant malaria among kala-azar patients is estimated to range between 4% and 61%.^56^ Hence, the perception of malaria evolving to kala-azar may origin from the standard clinical practice of ruling out malaria first.

The attitude of “doing everything” to get a positive kala-azar test further fuels the frequent belief that certain food would help to diagnose kala-azar. Although perhaps based on observations and experience, the belief reflects the hot–cold dichotomy in a healthy equilibrium state, a symbolic notion found in many cultures.^55,57^ People reported that the reason behind giving banana and chicken to a kala-azar–suspected patient is to amplify the hot power that is raging in the patient’s body—the heightened heat would surely make the diagnosis positive. It seems unusual to congratulate someone of being sick (with kala-azar), yet in this context, it symbolizes the end of an arduous journey and hopes that the cure is finally within reach.

Our study found a positive perception toward allopathic treatment: people are convinced that kala-azar needs to be ultimately treated by (medical) injection at the hospitals. Although acknowledged as “hard” medicine because of the side effects, they trust that the drug is making the patients well again, an essential factor for compliance.^58^ Negative perceptions prevail toward the quality of care that is provided at the public hospitals, primarily related to the limited resources such as the availability of the doctors and more importantly, the drug. In Rahad and Galabat localities, the recurrent stock-outs of the medicine were criticized as they can lead to interruption of treatment. Illicit trade in medicines adds to the dissatisfaction with the service, especially when they compare with centers/hospitals receiving external support from NGOs. This has led people to take the journey to these centers, or to Gadarif city, thus not necessarily utilizing the closest health facility.

An overarching barrier for accessing kala-azar care is the cost of an episode—being sick with kala-azar implies paying for multiple tests, treatments, and hospitalization. Financial constraints were given as the most plausible explanation for why kala-azar patients wait for weeks and months before going to the hospitals. The fact that the medicine is for free (and not the diagnostic tests) does not compensate for the considerable costs that the family has to spend from the pocket directly, and although we did not find evidence that treatment compliance is jeopardized, several coping mechanisms (such as borrowing or selling assets) were clearly in place. Financial barriers during the rainy season—due to increased transport cost related to the physical barriers—are particularly cumbersome for those without a stable income. Our findings are consistent with an earlier economic analysis of kala-azar in Sudan, which estimated the total cost at US$450 for an episode,^11^ totally unaffordable for most subsistence farmers in this area (61% of the population are reported to live below the poverty line of 250SDG [US$37] per month^59^). Although women need their husband’s permission to seek care in this culture,^28^ the delay is often due to lack of money. People also more readily travel to free-of-charge treatment centers or the ones known to offer more in-kind support (nutrition, etc.). However, the rigidly defined gender roles for women and their unequal access to resources compound more difficulties for women.

The participants indicated that some barriers have been and could be ameliorated through several interventions. Health education to address the misconceptions and to empower the community in negotiating access in the medical system is demanded. Although active case detection was deemed unnecessary in areas with good access to treatment centers,^23^ a more targeted approach toward villages with least access to services after the rainy/autumn season should help save lives. The poorest and those who live furthest away could benefit from a targeted support system, such as transport loan funds, health insurance, community loan funds, or charity such as the Islamic “zakat.” Quality of care should be standardized, through equitable distribution of resources to the hospitals. Health system strengthening efforts will benefit kala-azar patients through improvement of the supply system and offset the discrepancies between NGO–supported centers and public ones. Another recommendation that goes beyond the health sector is to enhance the propoor policies in Gadarif, and government to step up its actions in reducing poverty. The sustainability of access-to-care for kala-azar needs political and resources commitments.

One limitation of our study was that we did not fully capture the perspective of children and adolescents, who make up most kala-azar patients in Sudan. The group discussions were conducted in gender division, but the varied age in each group may limit the younger participants in expressing their views. We also could not explore fully the ethnic dimensions in the study as it was not possible to conduct the data collection and enroll participants along tribal lines in the current political climate in Sudan. We cannot exclude the social desirability bias in some of the responses, knowing the field team came from Gadarif. Although generalizability is chided as a limitation in a qualitative study,^57^ we believed that reasonable extrapolation of our findings is not impossible. The generalizability of this study does not derive mechanistically from the sample but from the concepts emerging from the findings (such as the delay in diagnosis or persistent economic burden) that may well be relevant to other settings or other health problems. The context of Gadarif is specific, yet the suffering is not.

We believe this study offers a timely thorough insight into the community perspective on kala-azar in this area and help explain the reasons behind the delay in seeking kala-azar care documented in quantitative surveys.^60,61^ It is essential for policymakers and other stakeholders to understand the barriers explored here as reducing delay would be contingent on addressing these. In regard to the technical tools, there has been progress in the recent decade, i.e., the RDT and a shorter treatment regimen, yet these are still far from optimal. Several factors revealed here merit further research, such as the diagnostic bottlenecks (lack of trust in RDT, an algorithm for prolonged fever such as kala-azar) and more empirical studies to measure the various dimensions of access over time that may predict health behavior, service utilization, and health outcomes. Last but not least, development of better vector control tools and other preventive measures is critical to lessen the kala-azar burden to the communities in the long run.

## CONCLUSION

Kala-azar is an infectious disease of poverty in southern Gadarif, Sudan. Despite allegedly more efforts to control the disease, the access to quality kala-azar care remains problematic as is observed for many other health conditions in such settings.^62^ To alleviate suffering, the multiple barriers they face should be considered before implementing any interventions. The financial accessibility should be prioritized through a multisectoral approach designed to have wider benefit for health for all.

## Supplementary Material

Supplemental Material.

## References

[1] World Health Organization, 2017 *Weekly Epidemiological Record*, Vol. 92. Geneva, Switzerland: World Health Organization. 557–572.

[2] World Health Organization, 2016 *Global Health Observatory* Available at: http://www.who.int/gho/neglected_diseases/leishmaniasis/en/. Accessed April 3, 2016.

[3] Chappuis F Sundar S Hailu A Ghalib H Rijal S Peeling RW Alvar J Boelaert M, 2007 Visceral leishmaniasis: what are the needs for diagnosis, treatment and control? Nat Rev Microbiol 5: S7–S16. 10.1038/nrmicro1748 17938629

[4] Medley GF Hollingsworth TD Olliaro PL Adams ER, 2015 Health-seeking behaviour, diagnostics and transmission dynamics in the control of visceral leishmaniasis in the Indian subcontinent. Nature 528: S102–S108. 26633763 10.1038/nature16042

[5] Peters DH Garg A Bloom G Walker DG Brieger WR Hafizur Rahman M, 2008 Poverty and access to health care in developing countries. Ann N Y Acad Sci 1136: 161–171. 17954679 10.1196/annals.1425.011

[6] Jacobs B Ir P Bigdeli M Annear PL Van Damme W, 2012 Addressing access barriers to health services: an analytical framework for selecting appropriate interventions in low-income Asian countries. Health Policy Plan 27: 288–300. 21565939 10.1093/heapol/czr038

[7] Alvar J Yactayo S Bern C. Leishmaniasis and poverty. Trends Parasitol 22: 552–557. 17023215 10.1016/j.pt.2006.09.004

[8] Diro E Lynen L Ritmeijer K Boelaert M Hailu A van Griensven J, 2014 Visceral leishmaniasis and HIV coinfection in east Africa. PLoS Negl Trop Dis 8: e2869. 24968313 10.1371/journal.pntd.0002869 PMC4072530

[9] Seaman J Mercer AJ Sondorp HE Herwaldt BL, 1996 Epidemic visceral leishmaniasis in southern Sudan: treatment of severely debilitated patients under wartime conditions and with limited resources. Ann Intern Med 124: 664–672. 8607595 10.7326/0003-4819-124-7-199604010-00007

[10] Al-Salem W Herricks JR Hotez PJ, 2016 A review of visceral leishmaniasis during the conflict in South Sudan and the consequences for east African countries. Parasit Vectors 9: 460. 27549162 10.1186/s13071-016-1743-7 PMC4994383

[11] Meheus F Abuzaid AA Baltussen R Younis BM Balasegaram M Khalil EA Boelaert M Musa AM, 2013 The economic burden of visceral leishmaniasis in Sudan: an assessment of provider and household costs. Am J Trop Med Hyg 89: 1146–1153. 24189368 10.4269/ajtmh.12-0585 PMC3854893

[12] Pascual Martínez F Picado A Roddy P Palma P, 2012 Low castes have poor access to visceral leishmaniasis treatment in Bihar, India. Trop Med Int Health 17: 666–673. 22385129 10.1111/j.1365-3156.2012.02960.x

[13] WHO, 2016 Leishmaniasis in high-burden countries: an epidemiological update based on data reported in 2014. Wkly Epidemiol Rec 91: 287–296. 27263128

[14] El-Hassan AM Zijlstra EE, 2001 Leishmaniasis in Sudan. Trans R Soc Trop Med Hyg 95 (Suppl 1): S27–S58. 11370250 10.1016/s0035-9203(01)90218-4

[15] Elnaiem DE, 2011 Ecology and control of the sand fly vectors of *Leishmania donovani* in east Africa, with special emphasis on *Phlebotomus orientalis*. J Vector Ecol 36 (Suppl 1): S23–S31. 21366778 10.1111/j.1948-7134.2011.00109.x

[16] Malaria Consortium, 2010 *Leishmaniasis Control in Eastern Africa: Past and Present Efforts and Future Needs. Situation and Gap Analysis*. Leeds, United Kingdom: COMDIS.

[17] Burki T, 2009 East African countries struggle with visceral leishmaniasis. Lancet 374: 371–372. 19655434 10.1016/s0140-6736(09)61401-x

[18] Ritmeijer K Melaku Y Mueller M Kipngetich S O’Keeffe C Davidson RN, 2006 Evaluation of a new recombinant K39 rapid diagnostic test for Sudanese visceral leishmaniasis. Am J Trop Med Hyg 74: 76–80. 16407349

[19] Musa A 2012 Sodium stibogluconate (ssg) & paromomycin combination compared to ssg for visceral leishmaniasis in east Africa: a randomised controlled trial. PLoS Negl Trop Dis 6: e1674. 22724029 10.1371/journal.pntd.0001674 PMC3378617

[20] World Health Organization, 2010 *Control of the Leishmaniases: Report of a Meeting of the WHO Expert Committee on the Control of Leishmaniases, March 22–26, 2010, Geneva*. Geneva, Switzerland: WHO.

[21] Adam GK Ali KM Abdella YH Omar SM Ahmed MA Abdalla TM Ali AA, 2016 Trend in cumulative cases and mortality rate among visceral leishmaniasis patients in eastern Sudan: a 14-year registry, 2002–2015. Int J Infect Dis 51: 81–84. 27596686 10.1016/j.ijid.2016.08.021

[22] Pigott DM 2014 Global distribution maps of the leishmaniases. eLife 3: e02851. 10.7554/eLife.02851 PMC4103681 24972829

[23] Mueller YK 2012 Burden of visceral leishmaniasis in villages of eastern Gedaref State, Sudan: an exhaustive cross-sectional survey. PLoS Negl Trop Dis 6: e1872. 23133683 10.1371/journal.pntd.0001872 PMC3487394

[24] Alvar J Vélez ID Bern C Herrero M Desjeux P Cano J Jannin J Boer M; WHO Leishmaniasis Control Team, 2012 Leishmaniasis worldwide and global estimates of its incidence. PLoS One 7: e35671. 22693548 10.1371/journal.pone.0035671 PMC3365071

[25] World Health Organization, 2015 *Visceral Leishmaniasis: Control Strategies and Epidemiological Situation Update in East Africa: Report of a WHO Bi-Regional Consultation Addis Ababa, Ethiopia, March 9–11, 2015*. Geneva, Switzerland: WHO.

[26] Atia AM 2015 Sodium stibogluconate and paromomycin for treating visceral leishmaniasis under routine conditions in eastern Sudan. Trop Med Int Health 20: 1674–1684. 26427033 10.1111/tmi.12603

[27] den Boer M Argaw D Jannin J Alvar J, 2011 Leishmaniasis impact and treatment access. Clin Microbiol Infect 17: 1471–1477. 21933305 10.1111/j.1469-0691.2011.03635.x

[28] Gerstl S Amsalu R Ritmeijer K, 2006 Accessibility of diagnostic and treatment centres for visceral leishmaniasis in Gedaref State, northern Sudan. Trop Med Int Health 11: 167–175. 16451340 10.1111/j.1365-3156.2005.01550.x

[29] Ibrahim ME 1999 Kala-azar in a high transmission focus: an ethnic and geographic dimension. Am J Trop Med Hyg 61: 941–944. 10674674 10.4269/ajtmh.1999.61.941

[30] Bucheton B Kheir MM El-Safi SH Hammad A Mergani A Mary C Abel L Dessein A, 2002 The interplay between environmental and host factors during an outbreak of visceral leishmaniasis in eastern Sudan. Microbes Infect 4: 1449–1457. 12475635 10.1016/s1286-4579(02)00027-8

[31] Thaddeus S Maine D, 1994 Too far to walk: maternal mortality in context. Soc Sci Med 38: 1091–1110. 8042057 10.1016/0277-9536(94)90226-7

[32] Aday LA Andersen R, 1974 A framework for the study of access to medical care. Health Serv Res 9: 208–220. 4436074 PMC1071804

[33] Andersen RM, 1995 Revisiting the behavioral model and access to medical care: does it matter? J Health Soc Behav 36: 1–10. 7738325

[34] Sudan Central Bureau of Statistic, 2008 *Sudan National Population and Housing Census* Available at: http://www.cbs.gov.sd/en/files.php?id=7#&panel1-5. Accessed August 1, 2016.

[35] Miller C, 2005 Power, land and ethnicity in the Kassala-Gedaref States. *Land, Ethnicity and Political Legitimacy in Eastern Sudan*. Le Caire: Cedej, 3–58.

[36] Miller C Manga AAA, 2005 The West African communities in Gedaref State: processes of settlement and local integration. Miller C, ed. *Land, Ethnicity And Political Legitimacy In Eastern Sudan*. Cairo, Egypt: CEDEJ, 375–424.

[37] Sudan Central Bureau of Statistics, 2009 *Sudan National Baseline Household Survey 2009* Available at: http://ghdx.healthdata.org/record/sudan-north-national-baseline-household-survey-nbhs-2009. Accessed August 1, 2016.

[38] Mays N Pope C, 2000 Assessing quality in qualitative research. BMJ 320: 50. 10617534 10.1136/bmj.320.7226.50 PMC1117321

[39] Braun V Clarke V, 2006 Using thematic analysis in psychology. Qual Res Psychol 3: 77–101.

[40] Braun V Clarke V, 2014 What can “thematic analysis” offer health and wellbeing researchers? Int J Qual Stud Health Well-being 9: 26152. 25326092 10.3402/qhw.v9.26152 PMC4201665

[41] Khatri RB Dangi TP Gautam R Shrestha KN Homer CSE, 2017 Barriers to utilization of childbirth services of a rural birthing center in Nepal: a qualitative study. PLoS One 12: e0177602. 28493987 10.1371/journal.pone.0177602 PMC5426683

[42] Phillips KA Morrison KR Andersen R Aday LA, 1998 Understanding the context of healthcare utilization: assessing environmental and provider-related variables in the behavioral model of utilization. Health Serv Res 33: 571–596. 9685123 PMC1070277

[43] Posse M Meheus F van Asten H van der Ven A Baltussen R, 2008 Barriers to access to antiretroviral treatment in developing countries: a review. Trop Med Int Health 13: 904–913. 18466183 10.1111/j.1365-3156.2008.02091.x

[44] Long Q Li Y Wang Y Yue Y Tang C Tang S Squire SB Tolhurst R, 2008 Barriers to accessing TB diagnosis for rural-to-urban migrants with chronic cough in Chongqing, China: a mixed methods study. BMC Health Serv Res 8: 202. 18828929 10.1186/1472-6963-8-202 PMC2567973

[45] Serizawa A Ito K Algaddal AH Eltaybe RAM, 2014 Cultural perceptions and health behaviors related to safe motherhood among village women in eastern Sudan: ethnographic study. Int J Nurs Stud 51: 572–581. 24054068 10.1016/j.ijnurstu.2013.08.007

[46] Nackers F 2015 Determinants of visceral leishmaniasis: a case-control study in Gedaref State, Sudan. PLoS Negl Trop Dis 9: 1–16. 10.1371/journal.pntd.0004187 PMC4636291 26544177

[47] Thornton S Wasan K Piecuch A Lynd L Wasan E, 2010 Barriers to treatment for visceral leishmaniasis in hyperendemic areas: India, Bangladesh, Nepal, Brazil and Sudan. Drug Dev Ind Pharm 36: 1312–1319. 20545513 10.3109/03639041003796648

[48] Ensor T Cooper S, 2004 Overcoming barriers to health service access: influencing the demand side. Health Policy Plan 19: 69–79. 14982885 10.1093/heapol/czh009

[49] Ahluwalia I 2003 Visceral leishmaniasis: consequences of a neglected disease in a Bangladeshi community. Am J Trop Med Hyg 69: 624–628. 14740879

[50] Alemu A Alemu A Esmael N Dessie Y Hamdu K Mathewos B Birhan W, 2013 Knowledge, attitude and practices related to visceral leishmaniasis among residents in Addis Zemen town, South Gondar, northwest Ethiopia. BMC Public Heal Heal 13: 382. 10.1186/1471-2458-13-382 PMC3642032 23617595

[51] Lopez-Perea N Sordo L Gadisa E Cruz I Hailu T Moreno J Aseffa A Cañavate C Custodio E, 2014 Knowledge, attitudes and practices related to visceral leishmaniasis in rural communities of Amhara State: a longitudinal study in northwest Ethiopia. PLoS Negl Trop Dis 8: e2799. 24743328 10.1371/journal.pntd.0002799 PMC3990515

[52] Elnaiem DE Mukhawi AM Hassan MM Osman ME Osman OF Abdeen MS Abdel Raheem MA, 2003 Factors affecting variations in exposure to infections by *Leishmania donovani* in eastern Sudan. East Mediterr Health J 9: 827–836. 15748079

[53] Ritmeijer K Davies C Van Zorge R Wang SJ Schorscher J Dongu’du SI Davidson RN, 2007 Evaluation of a mass distribution programme for fine-mesh impregnated bednets against visceral leishmaniasis in eastern Sudan. Trop Med Int Health 12: 404–414. 17313512 10.1111/j.1365-3156.2006.01807.x

[54] Kleinman A, 2010 Four social theories for global health. Lancet 375: 1518–1519. 20440871 10.1016/s0140-6736(10)60646-0

[55] Foster GM, 1976 Disease etiologies in non-western medical systems. Am Anthropol 78: 773–782.

[56] van den Bogaart E 2013 Concomitant malaria among visceral leishmaniasis in-patients from Gedarif and Sennar States, Sudan: a retrospective case-control study. BMC Public Health 13: 332. 23577673 10.1186/1471-2458-13-332 PMC3659061

[57] Mpanya A Hendrickx D Baloji S Lumbala C da Luz RI Boelaert M Lutumba P, 2015 From health advice to taboo: community perspectives on the treatment of sleeping sickness in the Democratic Republic of Congo, a qualitative study. PLoS Negl Trop Dis 9: e0003686. 25856578 10.1371/journal.pntd.0003686 PMC4391751

[58] Salih NA 2014 Liposomal amphotericin B for complicated visceral leishmaniasis (kala-azar) in eastern Sudan: how effective is treatment for this neglected disease? Trop Med Int Health 19: 146–152. 24433217 10.1111/tmi.12238

[59] World Bank, 2011 *A Poverty Profile of the Northern States of Sudan* Available at: http://siteresources.worldbank.org/INTAFRICA/Resources/257994-1348760177420/a-poverty-profile-for-the-northern-states-of-sudan-may-2011.pdf. Accessed September 1, 2017.

[60] KalaCORE, 2017 Cross-sectional Surveys In Bangladesh, India, Ethiopia & Sudan: Understanding Treatment Seeking & Household Economic Burden For VL Patients. Available at: http://www.kalacore.org/sites/default/files/content/resource/files/KalaCORE%20Survey%20WL6.pdf. Accessed June 15, 2017.

[61] KalaCORE, 2017 Visceral Leishmaniasis Treatment Access - The Reality On The Ground In Sudan. Available at: http://www.kalacore.org/sites/default/files/content/resource/files/The%20reality%20on%20the%20ground%20in%20Sudan%2 0_AtiaAlatiaby_WL6_2.pdf. Accessed June 15, 2017.

[62] Elmusharaf K Byrne E AbuAgla A AbdelRahim A Manandhar M Sondorp E O’Donovan D, 2017 Patterns and determinants of pathways to reach comprehensive emergency obstetric and neonatal care (CEmONC) in South Sudan: qualitative diagrammatic pathway analysis. BMC Pregnancy Childbirth 17: 278. 28851308 10.1186/s12884-017-1463-9 PMC5576292

