# Supplementary material for: “Kala-Azar is a Dishonest Disease”: Community Perspectives on Access Barriers to Visceral Leishmaniasis (Kala-Azar) Diagnosis and Care in Southern Gadarif, Sudan
Source: Am J Trop Med Hyg. 2018 Feb 26;98(4):1091–101. doi: 10.4269/ajtmh.17-0872 (PMC5928836; doi:10.4269/ajtmh.17-0872)
Supplement: Supplementary file 1 [file tpmd170872.SD1.pdf]

SUPPLEMENTAL MATERIALS

In-depth interview guide. *NOTE: This guide is to be used after proper solicitation of the information sheet and the informed consent is obtained*.

Visceral Leishmaniasis, also known as kala-azar, is a serious illness common in Gadarif. You have been contacted as you are living in areas where the disease can be found. We would like to ask you some questions about this and also discuss about experience, if you or someone that you know have had it before. We would like to understand better the people's opinion about the disease and challenges in getting care for this disease. The interview should take approximately 30–60 minutes to complete. PLEASE ALSO INTRODUCE YOURSELF.

Part A (this can be filled up at any moment)

1. Interviewer Name: ____
2. Translator: _____________________________- (if relevant)
3. Date of interview: ___/___/___
4. Village of interview: _________________ Locality_______________________
5. Time starting: ______________

Part B

1. Sex: M/F (circle)
2. Age: ___ (years)
3. Current occupation: ____
4. Tribe/ethnicity (if you think this is ok to ask, otherwise skip): ________________________________
5. How long have you lived in this village? _____ years _______months
6. Can you tell me something about yourself? (optional)

Part C: QUESTIONS

1. Can you tell me about your experience with kala-azar? (if you have)
2. In your opinion, what is kala-azar? (causes, transmission, symptoms, prevention . . .by asking: *What do you think causes kala-azar? How do people get it?*)
3. What is the specific name of kala-azar in your language?
4. a. How do you see kala-azar as compared with other diseases? (to know perception of severity)
   b. How do people in the village see it? (*How does it affect the community?*)
5. Who do you think can get kala-azar more? (*Who are more at risk for the disease?*)
6. What is generally done when a person gets kala-azar? (*What do people do when they think they have kala-azar? What do people do when they suffer from prolonged fever?*)
7. If people seek treatment of kala-azar, where do they go? Why? (*Beliefs and thoughts, preferences for healing/health-care services including perceptions of services rendered by different providers: traditional healers, hospitals* . . .)
8. If children or young people are sick, who made the decision to seek help? (Why?)
9. Can you tell me if getting care for kala-azar is easy? (*What made it difficult to get care on time for kala-azar?*)
10. Why do you think people with kala-azar sometimes come late to the hospital? (*Try to explore geographic accessibility: seasonal/farming activities, cultural/gender/age, administrative and financial barriers,* . . .)
11. What do you think can cure kala-azar?
12. Why do you go to health centers or hospitals? (explore positive or negative perception toward available care for kala-azar)
13. If someone gets kala-azar, how does it impact the family?
14. And what do you think can make the situation better for kala-azar for you? And for the community?

Closure: Thanks
Time taken to finish the interview: __________
NOTE:
